# Application of Intraoperative CT-Guided Navigation in Simultaneous Minimally Invasive Anterior and Posterior Surgery for Infectious Spondylitis

**DOI:** 10.1155/2017/2302395

**Published:** 2017-02-16

**Authors:** Meng-Huang Wu, Navneet Kumar Dubey, Ching-Yu Lee, Yen-Yao Li, Chin-Chang Cheng, Chung-Sheng Shi, Tsung-Jen Huang

**Affiliations:** ^1^Department of Orthopedic Surgery, Taipei Medical University Hospital, Taipei, Taiwan; ^2^Department of Orthopedics, School of Medicine, College of Medicine, Taipei Medical University, Taipei, Taiwan; ^3^Graduate Institute of Clinical Medical Sciences, College of Medicine, Chang Gung University, Taoyuan, Taiwan; ^4^Graduate Institute of Biomedical Materials and Tissue Engineering, College of Biomedical Engineering, Taipei Medical University, Taipei, Taiwan; ^5^Department of Orthopedic Surgery, Chang Gung Memorial Hospital, Chiayi, Taiwan; ^6^College of Medicine, Chang Gung University, Taoyuan, Taiwan

## Abstract

This study was aimed at evaluating the safety and efficacy of using intraoperative computed tomography- (iCT-) guided navigation in simultaneous minimally invasive anterior and posterior surgery for infectious spondylitis. Nine patients with infectious spondylitis were enrolled in this study. The average operative time was 327.6 min (range, 210–490) and intraoperative blood loss was 407 cc (range, 50–1,200). The average duration of hospital stay was 48.9 days (range, 11–76). Out of a total of 54 pedicle screws employed, 53 screws (98.1%) were placed accurately. A reduced visual analog scale on back pain (from 8.2 to 2.2) and Oswestry disability index (from 67.1% to 25.6%) were found at the 2-year follow-up. All patients had achieved resolution of spinal infection with reduced average erythrocyte sedimentation rate (from 83.9 to 14.1 mm/hr) and average C-reactive protein (from 54.4 to 4.8 mg/dL). Average kyphotic angle correction was 10.5° (range, 8.4°–12.6°) postoperatively and 8.5° (range, 6.9°–10.1°) after 2 years. In conclusion, the current iCT-guided navigation approach has been demonstrated to be an alternative method during simultaneous minimally invasive anterior and posterior surgery for infectious spondylitis. It can provide a good intraoperative orientation and visualization of anatomic structures and also a high pedicle screw placement accuracy in patient's lateral decubitus position.

## 1. Introduction

In recent years, many approaches for anterior spinal surgery have been developed to correct and treat injuries or lesions by providing adequate decompression and debridement, maintenance, and reinforcement of the stability [1, 2]. However, these anterior approaches faced the potential complications like vessel or nerve injuries due to a large incision and extensive anatomical dissection [3]. Furthermore, in order to overcome these limitations, posterior approaches were also developed which later were found to be associated with graft collapse or nonunion [4]. The drawback associated in these conventional methods has attracted attention towards minimally invasive spinal surgery (MISS) which provides minimized damage to paraspinal soft tissues and musculature thereby preserving tissue structures with highly reduced surgical complications. However, MISS may limit visualization and identification of anatomical landmarks during surgery due to smaller incisions and reduced soft tissue dissections that might lead to more severe complications. Therefore, in order to improve the identification of anatomic structure and the accuracy of pedicle screws placement, the intraoperative computed tomography- (iCT-) guided navigation has been developed and may play a significant role in MISS [5]. Webb et al. had a cadaveric study using a fluoroscopy-based navigation for minimally invasive direct lateral interbody fusion in which the navigation system had provided a high accuracy up to less than 1 mm over L2–L5 and largely reduced radiation exposure for surgeons [6]. This feasibility study had demonstrated the benefits and safety of navigation in the anterior spinal surgery. However, up to date, there is scarcity of data on simultaneous anterior and posterior minimally invasive approach using iCT-guided navigation. In this study, we aimed to evaluate the safety and efficacy by using iCT-guided navigation in simultaneous minimally invasive anterior and posterior surgery for infectious spondylitis.

## 2. Materials and Methods

### 2.1. Study Design and Patient Population

This retrospective cohort study included 9 patients (5 females, 4 males) with infectious spondylitis (Table 1). All the patients underwent simultaneous minimally invasive anterior and posterior surgery with iCT-guided navigation between May 2011 and May 2014. This study was approved by the institutional review board (IRB number 102-3501B). All demographic and perioperative data were collected from chart reviews and prospectively recorded preoperatively, postoperatively, and after 2-year follow-up in institutional Spine Operation Registry, including the following factors: sex, age, ASA classification, diagnosis, surgical level, operative time, blood loss, neurologic status as American Spinal Injury Association (ASIA) impairment scale, back pain score (visual analog scale, VAS), functional scale (Oswestry disability index, ODI), radiographic examination (kyphotic correction angle immediately after surgery and 2-year follow-up), inflammatory markers, and radiation dose.

### 2.2. Surgical Technique

A preoperative examination of patients including plain radiographs and magnetic resonance imaging (MRI), blood and urine cultures, and inflammatory status (erythrocyte sedimentation rate and C-reactive protein) was done and then simultaneous anterior and posterior spinal surgery was undertaken. Pedicle screw was first inserted with the aid of iCT-guided navigation in the lateral decubitus position followed by simultaneous minimal access spinal surgery (MASS) for anterior decompression and reconstruction. Finally, the connecting rods were inserted and anchored in pedicle screw heads to achieve stabilization.

**Figure 1:**
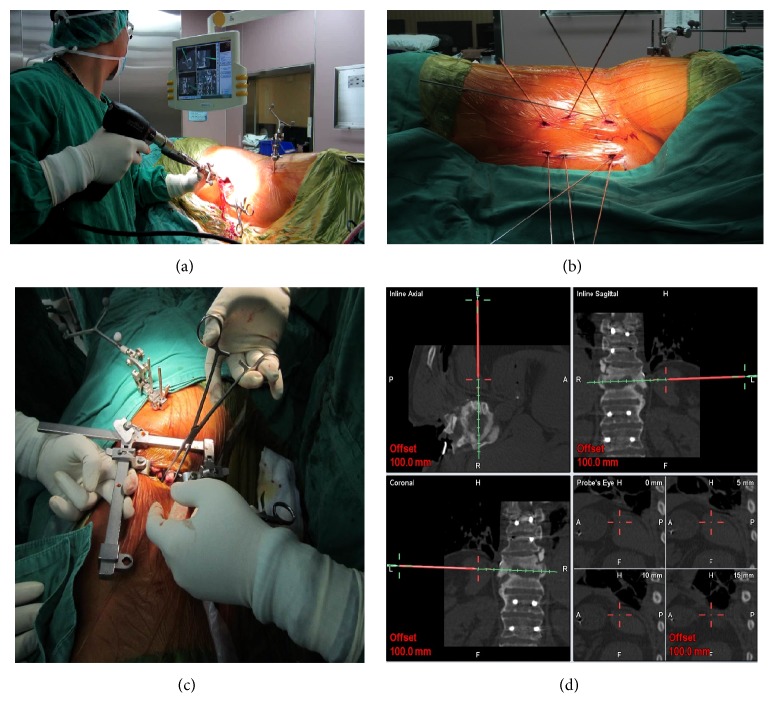
Intraoperative image demonstrating simultaneous anterior minimal access spinal surgery (MASS) and minimally invasive posterior spinal surgery. The trajectories of pedicle screws can be made by a registered drill guide and the iCT-guided navigation (a). The guidewire facilitates cannulated pedicle screw insertion (b). The consecutive anterior spinal surgery can be performed at the same position (c). The intraoperative navigation for MASS approach showing the site of infectious spondylitis and paraspinal structures (d).

**Figure 2:**
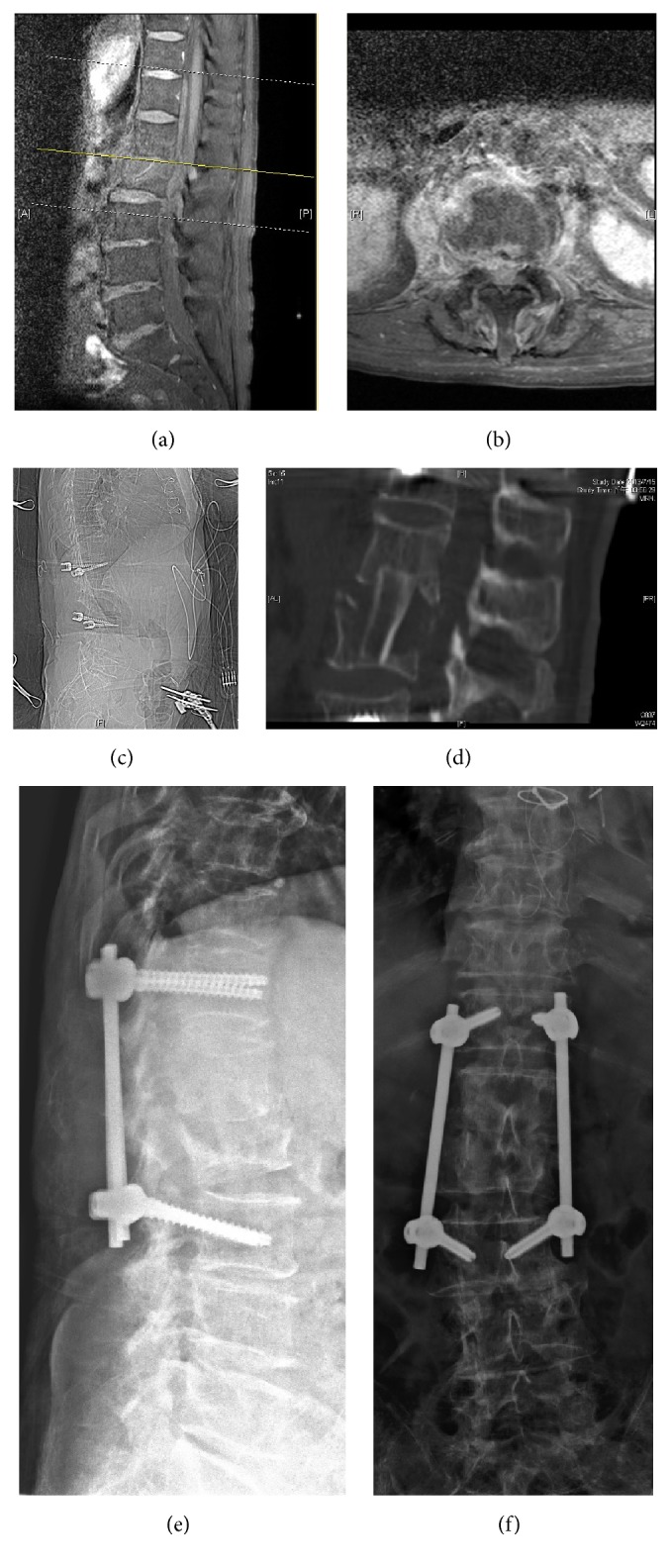
L1-L2 infectious spondylitis accompanied with epidural abscess treated with simultaneous minimally invasive anterior and posterior surgery using the iCT-guided navigation. Preoperative MRI demonstrated L1-L2 vertebral osteomyelitis and epidural abscess with obvious canal compromise (a, b). Intraoperative CT image obtained following simultaneous anterior and posterior spinal surgery (c, d) showing the spinal decompression and reconstruction. Partial resection of the L2 vertebra with reconstruction of L1-L2 with a percutaneous pedicle screw-rod construct and interbody iliac strut bone grafting (e, f).

As demonstrated in Figure 1, patient was placed in the lateral decubitus position to allow simultaneous approaches to the anterior and posterior spine. The entire lateral thoracoabdominal region was included in the operative field, including the iliac crest for autograft harvest when needed. The reference array was fixed to the iliac crest just above anterior superior iliac spine away from the planned bone graft harvest site. A registration CT scan was then performed and the image was transferred to the navigation system. CT images before and after screw insertion were reviewed for screw position accuracy. The radiation dose from CT was noted as effective dose from converting the total dose length product with a conversion factor for the trunk region (0.015) [7]. We started the surgery from posterior instrumentation using percutaneous MISS approach. After identifying the pedicle entry, the screw tracks were then prepared with a registered drill guide. Guide wires were inserted into the tracks as guides. After all the pedicle screw tracks were prepared, cannulated pedicle screws of sufficient length and diameter were placed in the planned screw track to the optimal depth by the assistance of the navigation system. After the screw had been inserted, a confirmatory CT scan was immediately done as a second registration CT scan for anterior MASS. If screw malposition was seen on the confirmation CT, then the malpositioned screws were immediately removed. The final definite fixation with rods was done after anterior reconstruction with bone graft. MASS techniques had previously been described in detail [8, 9]. A summary of the MASS for the treatment of target lesion included curettage and debridement of the lesion site, obtaining tissue specimens, decompression of the epidural space, and placement of autogenous bone struts from the ilium or excised rib into the intervertebral space. After the completion of decompression and reconstruction, a second confirmatory CT scan was again performed to determine the correct placement of the bone graft. With MASS, there are three primary ways to access anterior spine lesions localized by iCT-guided navigation perioperatively, and all use a 2- to 3-inch skin incision for both thoracic and lumbar lesions. For thoracic spine lesions, a transthoracic anterolateral approach can be performed after an underlying rib is resected. A 28- or 32-French chest tube was placed at the end of surgery. For the thoracic-lumbar junction, a retropleural and retroperitoneal approach with only diaphragmatic crural detachment and no take-down procedure was used. A chest tube was needed instead of a postoperative Hemovac drainage if air leakage is present during normal saline filling of the retropleural space following the resumption of two-lung ventilation [8]. For the lumbar spine, exposure to the anterior lumbar spine is through a retroperitoneal method, and the wound was closed after insertion of a 1- and 8-inch Hemovac drain (Figure 2). The postoperative drain was removed when the drainage was <50 mL per 8 hours [9].

### 2.3. iCT-Guided Navigation System and Evaluation of Screw Positioning

The navigation system (Spine & Trauma iCT Navigation SW, Brainlab AG, Feldkirchen, Germany) consisted of a sliding gantry 24-slice CT scanner (SOMATOM Sensation, Siemens, Munich, Germany) with the following specifications: 120 peak tube voltage (kVp), rotation time of 1 second, multiplanar reconstructions with slice thickness and increment of 1.5 mm, and a frameless infrared-based navigation station (VectorVision Sky, Brainlab AG, Munich, Germany). Postoperative iCT images were reviewed for screw position using digital image measurements (Centricity PACS 3.0, GE Healthcare, Fairfield, CT, USA). The assessment used a measurement scale in the digital image system as described by Gertzbein and Robbins [10]. The pedicle encroachment with ≤2 mm was considered within safe zone while >2 mm was regarded as malpositioned and potentially unsafe. The malposition of the screw was evaluated by Kast criteria [11].

## 3. Results

The average operative time in the simultaneous anterior and posterior surgery was 327.6 minutes (range 210–493). The average blood loss during surgery was 407 cc (range, 50–1,200). The average duration of hospital stay was 48.9 days (range, 11–76). Furthermore, the average effective dose of radiation exposure during surgery was 15.4 millisieverts (mSv) (range, 19.4–26.8). The screw placement accuracy in this approach showed that, out of 54 pedicle screws employed, 53 screws were placed correctly (98.1%). The malpositioned screw was major breach as medial perforation with narrowing of the vertebral channel more than 25%.

Furthermore, following the surgery, the infection was controlled among all 9 patients treated with antibiotics, while one patient suffered from pneumonia, a major postoperative complication, and another patient was kept under extensive observation under intensive care unit due to delayed recovery from anaesthesia. We then evaluated the pain score on VAS which was 8.2 before surgery and 2.2 after 2-year follow-up. Besides, we also recorded the ODI score, a measure of functional improvement and recovery. The average preoperative ODI was 67.1 (range: 54.3–88.9) versus 25.6 (range: 11–40) postoperatively. As measured from radiograph, the average preoperative Cobb's kyphotic angle was 10.5° (range, 8.4°–12.6°) which decreased significantly to 8.5° (range, 6.9°–10.1°) after 2 years. The surgery also improved clinical and neurologic status according to ASIA impairment scale in which 4 patients showed improvement (Table 2). The posttreatment examination of the blood tests showed that the inflammatory markers including CRP and ESR were significantly reduced to 4.8 mg/dL (1.3–11) and 14.1 mm/hr (5–24) from preoperative value of 54.4 mg/dL (25–78) and 83.9 mm/hr (30–150) respectively.

In our study, 2 patients were identified with epidural abscess; one had psoas muscle abscess while the other one had lung empyema. With the aid of iCT-guided navigation, location of infected tissues can be accurately visualized which could help in debridement of infected tissues and draining of abscess.

## 4. Discussion

The major aims of surgical treatment in spinal infection are removal of infected tissues, reduction in kyphotic deformity, and pain thereby providing spinal stability [12, 13]. For the treatment of spinal disorders, the anterior approach has commonly been used as it provides an excellent decompression of the spinal cord; however, it often does not allow the adequate stabilization of the thoracic spine due to the normal kyphotic curvature. The anterior surgical approach in vertebral infection has also been reported to provide direct access to debride infected tissues [14]. However, only partial spinal stability can be achieved through anterior approach. Therefore, the addition of posterior approach may be helpful to correct kyphotic deformity and hence the spinal stability. Besides, both the anterior and posterior surgical approach have been reported with increased rate of misplaced screws [15] and vascular complications [16].

Based on this clinical study on surgical treatment of infectious spondylitis, we suggest that a simultaneous anterior and posterior MISS with iCT-guided navigation may overcome the limitations posed in either anterior or posterior approach and rendering complete circumferential decompression and stabilization. In the cadaveric study by Webb et al., the use of fluoroscopy-guided navigation for the anterior spinal surgery was feasible with an accuracy of less than 1 mm for L2–L5 [6]. Furthermore, the pedicle screw placement through the intraoperative cone-beam CT-guided navigation had been reported with higher accuracy than under fluoroscopic guidance in a prospective comparison study [17]. The iCT-guided navigation used in this approach also provided potential benefit to surgeon in avoiding radiation exposure which could not be possible in fluoroscopically assisted spinal surgery [18]. In a report, Ozturk et al. had also documented that simultaneous anterior and posterior spinal fusion surgery is faster with lesser blood loss and fewer complications when compared to sequential one-stage combined anterior and posterior spinal surgery [19]. This technique can be performed with the patients kept in the lateral decubitus position and can also facilitate the identification of anatomic structure and the screw implantation with a higher accuracy [5]. Moreover, the trajectory of the screw can also be analyzed immediately along with the length and position through confirmatory intraoperative CT scan [20]. Of note, the intraoperative localization of abscesses in epidural space and psoas muscles is still a challenge especially in minimally invasive spinal surgery [21]; however, iCT-guided navigation used in our study enabled localization of epidural and psoas muscle abscesses which were drained off and infected tissue was thoroughly debrided. So, the real-time visualization of vital organs and vessels through iCT-guided navigation during surgical process offers high safety. Based on overall treatment outcomes, we demonstrated that iCT-guided navigation could be applied safely during simultaneous minimally invasive anterior and posterior surgery for infectious spondylitis.

The limitations of this study include limited case numbers, a single surgeon experience, heterogeneous pathogens, and no comparative study. Furthermore, this report only included thoracic and lumbar spinal infections. However, the further detailed investigations by increasing number of cases and pathogen-based comparative study are needed to elucidate the potential benefits of this approach in spinal infections.

## 5. Conclusion

The current iCT-guided navigation approach has been demonstrated to be an alternative method during simultaneous minimally invasive anterior and posterior surgery for infectious spondylitis. It can provide a good intraoperative orientation and visualization of anatomic structures and also a high pedicle screw placement accuracy in patient's lateral decubitus position.

## Figures and Tables

**Table 1:** Demographics of simultaneous minimally invasive anterior and posterior spinal surgery for infectious spondylitis.

Sex		
Male		4
Female		5
Age		71 (50–79)
ASA classification		
2		2
3		6
4		1
Surgical level		
T10-T11		2
T11-L1		3
L1-L2		4
Causative pathogens		
*Staphylococcus aureus*		4
*Candida albicans*		1
*Candida tropicalis*		1
*Mycobacterium tuberculosis*		1
*Salmonella enterica*, serotype D		2

Laboratory tests	Pretreatment	Posttreatment

CRP (mg/dL)	54.4 (25–78)	4.8 (1.3–11)
ESR (mm/hr)	83.9 (30–150)	14.1 (5–24)

Functional scales	Preoperative	Postoperative (2 yr)

Visual analog scale	8.2 (7–10)	2.2 (1–3)
Oswestry disability index	67.1 (54.3–88.9)	25.6 (11–40)

	Postoperative	Postoperative (2 yr)

Kyphotic angle correction	10.5° (8.4°–12.6°)	8.5° (6.9°–10.1°)

ASA: American Society of Anesthesiologists; CRP: C-reactive protein; ESR: erythrocyte sedimentation rate.

**Table 2:** Preoperative and postoperative evaluation of improvement in American Spinal Injury Association (ASIA) impairment scale. Table showing change in the ASIA impairment scale between the preoperative status (vertical) and postoperative status at 2-year follow-up (horizontal).

Pre	Post			
	B	C	D	E
B	0^*∗∗*^	2^*∗∗∗*^	0^*∗∗∗*^	0^*∗∗∗*^
C	0^*∗*^	0^*∗∗*^	1^*∗∗∗*^	0^*∗∗∗*^
D	0^*∗*^	0^*∗*^	1^*∗∗*^	1^*∗∗∗*^
E	0^*∗*^	0^*∗*^	0^*∗*^	4^*∗∗*^

^*∗*^Poor; ^*∗∗*^similar; ^*∗∗∗*^improved.
